# Adipose-derived stem cells combined with platelet-rich plasma enhance wound healing in a rat model of full-thickness skin defects

**DOI:** 10.1186/s13287-021-02257-1

**Published:** 2021-04-06

**Authors:** Xuejun Ni, Xiuying Shan, Lili Xu, Wenjun Yu, Mingliang Zhang, Chen Lei, Nating Xu, Junyu Lin, Biao Wang

**Affiliations:** 1 Department of Plastic Surgery, the First Affiliated Hospital of Fujian Medical University, Fuzhou, 350005 China; 2 Department of Thyroid and Breast Surgery, the First Affiliated Hospital of Fujian Medical University, Fuzhou, 350005 China

**Keywords:** Adipose-derived stem cell, Platelet-rich plasma, Diabetic wound healing, Neovascularization

## Abstract

**Background:**

Wound healing is impaired in patients with diabetes due to the multifactorial etiology of the disease, which limits the therapeutic efficacy of various approaches. This study hypothesizes that the combination of adipose-derived stem cells (ADSCs) and platelet-rich plasma (PRP) might achieve optimally efficient diabetic wound healing.

**Methods:**

ADSCs were isolated from the adipose tissues of Sprague-Dawley (SD) rats. PRP was prepared by using a two-step centrifugation technique. A diabetic wound model was established on the backs of SD rats to evaluate the effect of ADSCs incorporated into PRP. Hematoxylin and eosin staining, immunofluorescence, and immunohistochemistry were performed to observe the changes in neovascularization. ELISA and Western blot were utilized to detect the angiogenesis-related protein expression levels. The proliferation of endothelial cells was assessed by the MTS assay.

**Results:**

ADSCs incorporated into PRP induced a higher wound closure rate than ADSCs, PRP, and negative control. The expression levels of VEGF, p-STAT3, and SDF-1 in the ADSC+PRP group were higher than those in the other groups. Moreover, the proliferation of endothelial cells was strongly stimulated by treatment with the combination of ADSC-conditioned medium (ADSC-CM) and PRP.

**Conclusions:**

PRP enhanced diabetic wound healing induced by ADSCs, and its promoting effect involved neovascularization.

## Background

Wound healing is a sequential and overlapping biological process that includes hemostasis, inflammation, growth, reepithelialization, and remodeling. It involves spatial and temporal coordination of multiple cell types and signaling pathways [1]. Accordingly, some injuries or disease states, such as diabetes, interrupt the normal wound healing progress and ultimately result in chronic or nonhealing wounds. Impairment of physiological activities in diabetes delays wound healing and ultimately results in chronic wounds. As the worldwide prevalence of diabetes mellitus and the life expectancy of diabetic patients have increased, the incidence of diabetic-related chronic wounds, which have become a serious public health problem, has also been amplified [2]. The development of efficient and accessible treatments for chronic wound healing is necessary.

Stem cell therapy might be a promising treatment option for chronic wounds due to the ability of stem cells to self-renew and differentiate into numerous types of cells [3]. It has been proven that stem cell therapy can contribute to diabetic wounds and other diseases [4–6]. Compared with other types of stem cells, adipose-derived stem cells (ADSCs) have specific advantages and fewer limitations; they are abundant and easily harvested, can be delivered via a minimally invasive procedure, are associated with fewer ethical issues, and have less risk of inducing tumorigenicity and the host immune response [7, 8]. A large number of studies have verified the therapeutic efficacy of ADSCs for wound healing [9–13]. However, the repair function of ADSCs is inherently restricted by the hostile microenvironment of chronic wounds due to their complex mechanism [14, 15].

Platelet-rich plasma (PRP), which provides an abundance of nutrients and offers a suitable microenvironment for ADSCs to promote proliferation and migration, is a good candidate for enhancing the wound healing effect of ADSCs due to its unique composition [16, 17]. PRP is an autologous product of blood plasma with a high platelet concentration and contains various growth factors and cytokines, such as platelet-derived growth factor (PDGF), transforming growth factor (TGF), vascular endothelial growth factor (VEGF), and insulin-like growth factor (IGF) [18]. Although more evidence of the efficacy of PRP alone as a treatment for wound healing is needed, the positive impact of PRP on chronic wounds has been proven in many studies [19]. More importantly, PRP can promote the proliferation and migration of ADSCs, which indicates that it has a potential synergistic regenerative effect on chronic wounds [20–22].

To our knowledge, relevant research on the combination of ADSCs and PRP for the treatment of wound healing is very limited. Based on the above results, we believe that the application of ADSCs in combination with PRP can improve the efficiency of wound healing. We established a wound model in diabetic rats and evaluated the synergistic effect of ADSCs and PRP on wound healing. The results showed that compared with the negative control, ADSCs, and PRP, the combination of ADSCs and PRP, which accelerated angiogenesis, greatly promoted wound healing in diabetic rats. The results of our study extend our understanding of the mechanism underlying the promoting effect of ADSCs and PRP on wound healing and might provide a constructive foundation for further research.

## Methods

### Isolation and culture of ADSCs

Male Sprague-Dawley (SD) rats were purchased from the Experimental Animals Centre of Fujian Medical University. All animal experiments were approved by the Animal Ethics Committee of the First Affiliated Hospital of Fujian Medical University. Rat ADSCs were isolated from the inguinal fat tissue of SD rats (20–24 weeks old, male) according to a previously reported method [23]. In brief, the inguinal fat tissues were isolated and washed with phosphate-buffered saline (PBS; Invitrogen, Carlsbad, CA). Then, the tissues were minced and digested with 0.1% collagenase type I (Sigma-Aldrich, St. Louis, MO) for 1 h at 37 °C. Enzyme activity was neutralized with low-glucose Dulbecco’s modified Eagle medium (LG-DMEM; HyClone, Logan, UT). Neutralized cells were centrifuged at 1000 rpm for 5 min. After centrifugation, the pellet was resuspended in LG-DMEM supplemented with 10% fetal bovine serum (FBS; Gibco, Carlsbad, CA). The isolated cells (2000 cells/cm^2^) were maintained in tissue culture flasks with culture medium at 37 °C in a 5% CO_2_ incubator. Fresh culture medium was changed every 2–3 days; cells were passaged upon reaching 95% confluency by trypsinization and propagated through passage 2.

### Confirmation of mesenchymal stem cell (MSC) characteristics

Immunophenotyping was performed by flow cytometry. Briefly, passage 3 ADSCs were harvested, washed with PBS, and incubated at 4 °C for 30 min in dark with fluorescein isothiocyanate (FITC)-labeled anti-CD34, FITC-labeled anti-CD45, FITC-labeled anti-CD90, and FITC-labeled anti-CD29 antibodies (BioLegend, San Diego, CA). Cells were stained with fluorescence-conjugated nonspecific IgG and IgG1 κ (BioLegend, San Diego, CA) to assess background fluorescence. The cells were analyzed using a FACSCalibur cytometer (Becton Dickinson, San Diego, CA), and FlowJo software V10 was used to generate histograms.

For adipogenic and osteogenic differentiation, assays were performed according to the manufacture’s instructions (Cyagen, Guangzhou, CHN). Passage 3 ADSCs reached 80% confluence, the medium was replaced with adipogenic differentiation medium A for 3 days, then medium B was replaced for 24 h alternately. After about 21 days of incubation, cells were fixed with 50% ethanol for 5 min, and oil red O staining was performed to confirm the formation of lipid droplets. Passage 3 ADSCs reached 70% confluence, and the medium was replaced with osteogenic reagents every 3 days. After 21 days of incubation, the presence of calcium nodules was evaluated by Alizarin red staining. Finally, the stained cells were photographed under a microscope (Olympus Microscopes, Tokyo, Japan).

### Preparation of PRP

PRP was obtained from the blood drawn from SD rats by cardiac puncture using a two-step centrifugation process according to a previously reported method [24]. The whole blood was centrifuged at 160×*g* for 20 min at room temperature to separate the buffy coat. The buffy coat was pipetted out and transferred into a sterile vacuette without citrate. The pipetted material was centrifuged again at 400×*g* for 15 min to separate the PRP. A thrombin activator (500 U bovine thrombin in 1 ml 10% calcium chloride) was added to activate the PRP.

### Transwell migration assay

To examine ADSC migration induced by different concentrations of PRP, Transwell Permeable Supports (#3422, CORNING, NY, USA) were applied. 2 × 10^4^ ADSCs were placed in the upper chamber of a Transwell plate, while 500 μl medium with different concentrations of PRP was added to the lower chamber. The concentration gradient of PRP was as follows: 0%, 5%, 10%, 20%, 30%, 40%, 80%, and 100%. LG-DMEM containing 10% FBS was added as a control. After incubation for 24 h at 37 °C, the migrated ADSCs were fixed with 4% paraformaldehyde and then stained using crystal violet. Finally, the stained cells were photographed under a microscope (Olympus Microscopes, Tokyo, Japan) and counted using ImageJ software (NIH, Bethesda, MD).

### Diabetic wound model and wound healing assessment

**Fig. 1:**
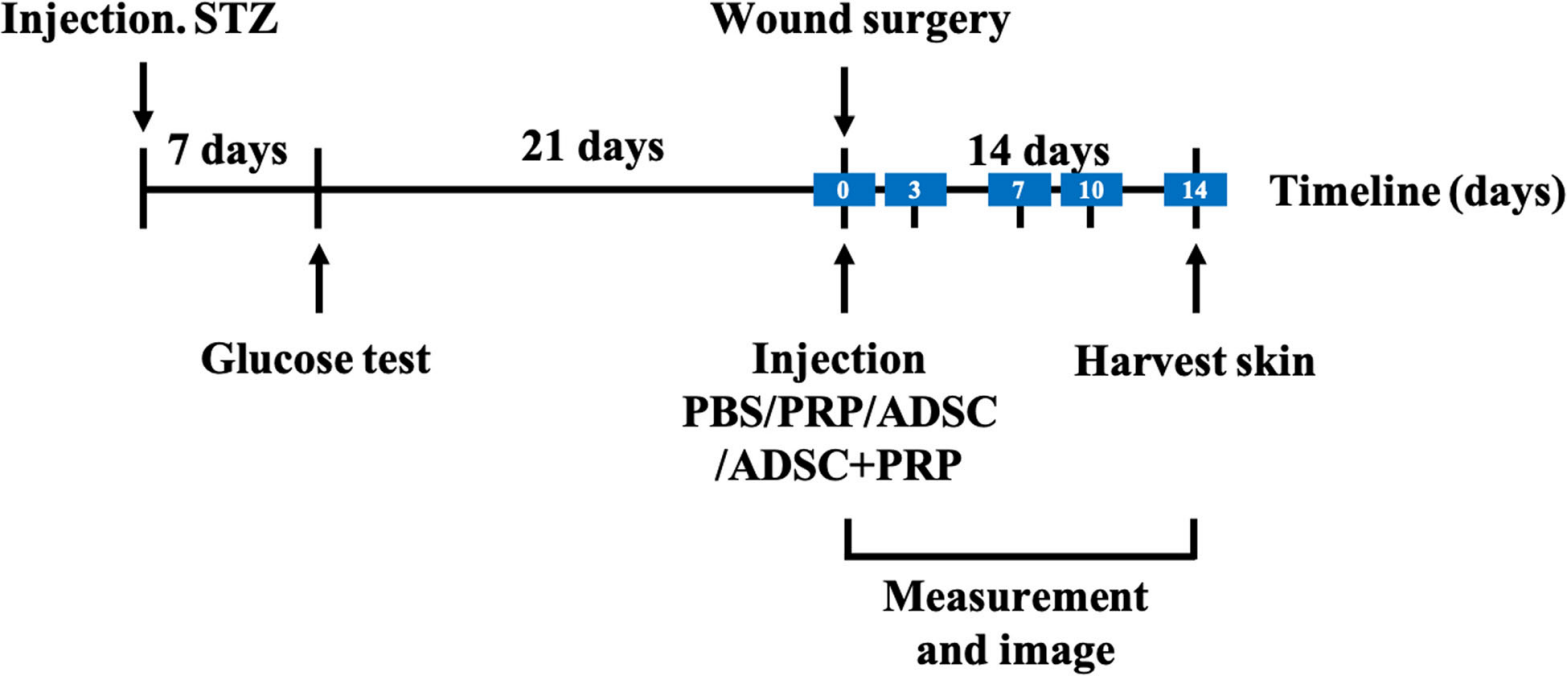
Timeline of the experimental design. PBS, PRP, ADSCs, or ADSC+PRP was injected into the wound area, and the wound size was measured and digitally photographed each day until day 14

Female SD rats (16 weeks) were injected a single dose with streptozotocin (50 mg/kg, Sigma-Aldrich, St. Louis, MO) to induce diabetes. The rats that exhibited hyperglycemia within 1 week after injection were confirmed as diabetic rats (blood glucose levels > 300 mg/dl), and the rats were then anesthetized by intraperitoneal injection of 10% chloral hydrate. The dorsal hair was removed, two circular wounds (diameter, 10 mm) were created on the dorsum of rats, and silicone rings were sutured around the wounds to prevent contracture. Rats were randomized to four groups receiving different treatments including PBS (negative control), ADSCs (positive control), PRP (positive control), and ADSC+PRP (*n* = 12 animals per group, 24 wounds per group). A total of 0.4 ml PBS (control), 0.4 ml ADSCs (1 × 10^6^/ml), 0.4 ml PRP, or 0.4 ml ADSCs (1 × 10^6^/ml) + PRP were injected subdermally at the four quadrants of each wound of the rats from each group respectively. Wounds were dressed with sterile Tegaderm dressing (3M Healthcare, St Paul, MN), which was changed every other day until wound closure. Wound size was measured from digital photographs 0, 1, 3, 7, 10, and 14 days after the operation and quantified using ImageJ software (NIH, Bethesda, MD). The experimental design is shown in Fig. 1.

### Histological observation

The tissue samples harvested on day 1, 3, 7, 10, and 14 postwounding were fixed in 4% paraformaldehyde overnight at 4 °C. After being washed with PBS, they were dehydrated in a graded ethanol series (30, 50, 70, 80, 90, and 100%), xylene, and paraffin washes and embedded in paraffin. Sections (4- to 6-mm-thick) were prepared from the paraffin-embedded wound tissues and then stained with hematoxylin and eosin as well as Masson’s trichrome (Yuanye, Shanghai, CHN). Tissue sections were observed using a microscope (Olympus Microscopes, Tokyo, Japan).

### Immunohistochemistry and immunofluorescence

The sections were prepared as described above. For immunohistochemistry, sections were deparaffinized in xylene and dehydrated through a graded series of alcohol. High-pressure antigen retrieval was performed with citrate antigen repair solution and then incubated in 3% hydrogen peroxide at room temperature for 20 min. The slices were incubated with primary rabbit polyclonal antibodies against stromal cell-derived factor-1 (SDF-1, 1:200 dilution, Abcam, Cambridge, MA) at 4 °C overnight and then incubated with a horseradish peroxidase-labeled secondary antibody at 37 °C for 30 min. Next, 3,3′-diaminobenzidine (DAB) was added at room temperature for 10 min, and the slices were then stained with hematoxylin at room temperature for 2 min. Finally, the slices were gently washed with deionized water, dehydrated in gradient alcohol solutions, mounted with neutral balsam, and observed using an optical microscope. Anti-CD31 and anti-CD34 antibodies (1:200 dilution, Abcam, Cambridge, MA) were used for immunofluorescence. As for immunofluorescence, the slices were incubated with the abovementioned primary antibodies at 4 °C overnight and then incubated with a secondary antibody at 37 °C for 1 h. The slices were finally stained with 4′,6-diamidino-2-phenylindole (DAPI, Invitrogen, Carlsbad, CA) and observed under a Zeiss LSM 700 confocal fluorescence microscope (ZEISS, Germany).

### Enzyme-linked immunosorbent assay (ELISA)

To measure the concentration of VEGF in wound tissues, the specimens were ground, trypsinized, and centrifuged to prepare protein extracts. The concentration of VEGF was then determined by an ELISA kit (R&D Systems, Minneapolis, MN) according to the manufacturer’s instructions. Briefly, 96-well plates were coated with a VEGF antibody. Two hundred microliters of standard, control, or sample was added to consecutive wells and incubated at room temperature for 2 h. Each well was then washed 3 times with wash buffer, and 200 μl VEGF conjugate was added to each well. After incubation at room temperature for 2 h, 200 μl substrate solution was added to each well, and the samples were incubated for 20 min at room temperature. The concentrations were then determined at 450 nm using a microplate reader. VEGF concentrations were calculated from the standard curve.

### Western blot

Protein concentration was quantified using the BCA method. Western blotting was carried out according to the standard protocols. Proteins were separated by 10% SDS-PAGE and transferred to a PVDF membrane. The membrane containing transferred proteins was blocked with 5% skim milk in TBS at room temperature for 1 h and with 1:1000 dilutions of anti-p-STAT3 (Abcam, Cambridge, MA) overnight at 4 °C. The next day, the membranes were incubated with secondary antibody at a 1:1000 dilution for 2 h at room temperature after washed with TBST for three times. Chemiluminescence detection was performed using the ECL reagent (Bio-Rad Laboratories). The strength of the signal for each protein was determined based on the corresponding band intensity of the scanned image.

### Endothelial cell culture and the MTS assay

Rat dermis microvascular endothelial cells were isolated from dermal microvasculature and cultured as previously described [25]. To evaluate the proliferation of viable endothelial cells, a total of 5 × 10^3^ endothelial cells per well were cultured in 96-well plates in 100 μl medium with or without high glucose (HG; 25 mmol/l, final concentration), which contained ADSC culture medium (ADSC-CM), 20% PRP, ADSC-CM + 20% PRP or 10% FBS as the control. Cell proliferation was assessed using The CellTiter 96 AQueous Non-radioactive Cell Proliferation Assay following the manufacturer’s instructions (Promega, Madison, WI). Twenty microliters of 3-(4,5-dimethylthiazol-2-yl)-5-(3-carboxymethoxyphenyl)-2-(4-sulfophenyl)-2H-tetrazolium (MTS, Promega, Madison, WI) was added to each well. After incubation for 4 h, absorption values were measured at a wavelength of 490 nm using a microplate reader.

### Statistical analysis

Statistical analysis was performed with IBM SPSS Statistics 25. All data were expressed as the mean ± SEM. One-way ANOVA analysis of variance for multiple comparisons with Tukey’s post hoc test was used to determine statistical significance, which was defined by a *p* value < 0.05 (significance was set to *p < 0.05, **p < 0.01, and ***p < 0.001).

## Results

### Characterization and identification of ADSCs

**Fig. 2:**
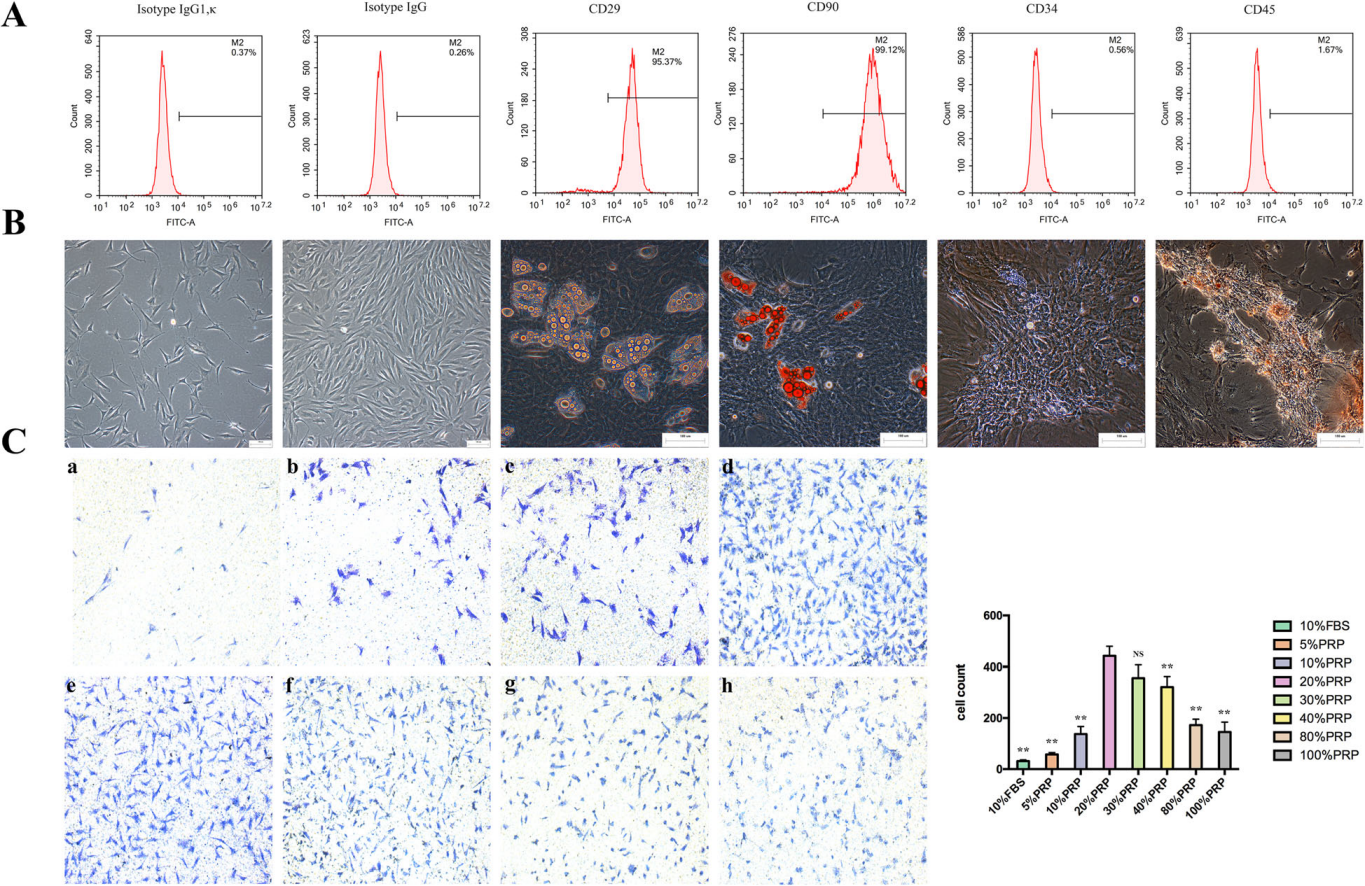
Characterization of ADSCs and the optimal concentration of PRP for combination with ADSCs. **a** Immunophenotypical analysis of ADSCs by flow cytometry. Passage 3 ADSCs, which were positive for CD29 and CD99 and negative for CD34, expressed typical MSC surface antigens. **b** Multipotent differentiation properties of ADSCs. Passage 3 ADSCs were cultured in adipogenic and osteogenic differentiation media for 21 days. Then, differentiation was evaluated by staining lipid droplets with oil red O (adipogenic, right), and calcium nodules were detected by Alizarin red staining (osteogenic, left). Representative images of 3 independent experiments are shown. Scale bars = 100 μm. **c** Migration efficiency was evaluated by crystal violet staining and microscopy and quantified with ImageJ. The analysis generally suggested that the optimal concentration of PRP in combination with ADSCs was 20%, with efficiencies ranging from 5 to 100%. All experiments were performed in triplicate and were repeated three times to confirm the findings (*p < 0.05). One-way ANOVA and Tukey’s post hoc test showed statistically significant differences overall between the eight groups. Values were expressed as mean ± SEM (*n* = 5 high-powered fields per well, three wells per group). Significance was set to *p < 0.05, **p < 0.01, and ***p < 0.001

The MSC characteristics of isolated ADSCs were first confirmed based on the expression of two MSC surface antigens (CD29 and CD90) and two endothelial progenitor cell markers (CD34 and CD45), as measured by flow cytometry. Positive expression of CD29 and CD90 and negative expression of CD34 and CD45 were detected, validating the identity of the ADSCs used in this study (Fig. 2a). In addition, we tested the multilineage differentiation potential of ADSCs. Oil red O staining demonstrated that lipid droplet formation was induced by adipogenic medium in ADSCs after 21 days, and Alizarin red staining showed calcium nodules in ADSCs after 21 days of induction with osteogenic medium (Fig. 2b). Thus, the ADSCs utilized in this study were successfully isolated, and their multilineage potential was confirmed.

### Combined treatment with ADSCs and PRP improved diabetic wound healing

**Fig. 3:**
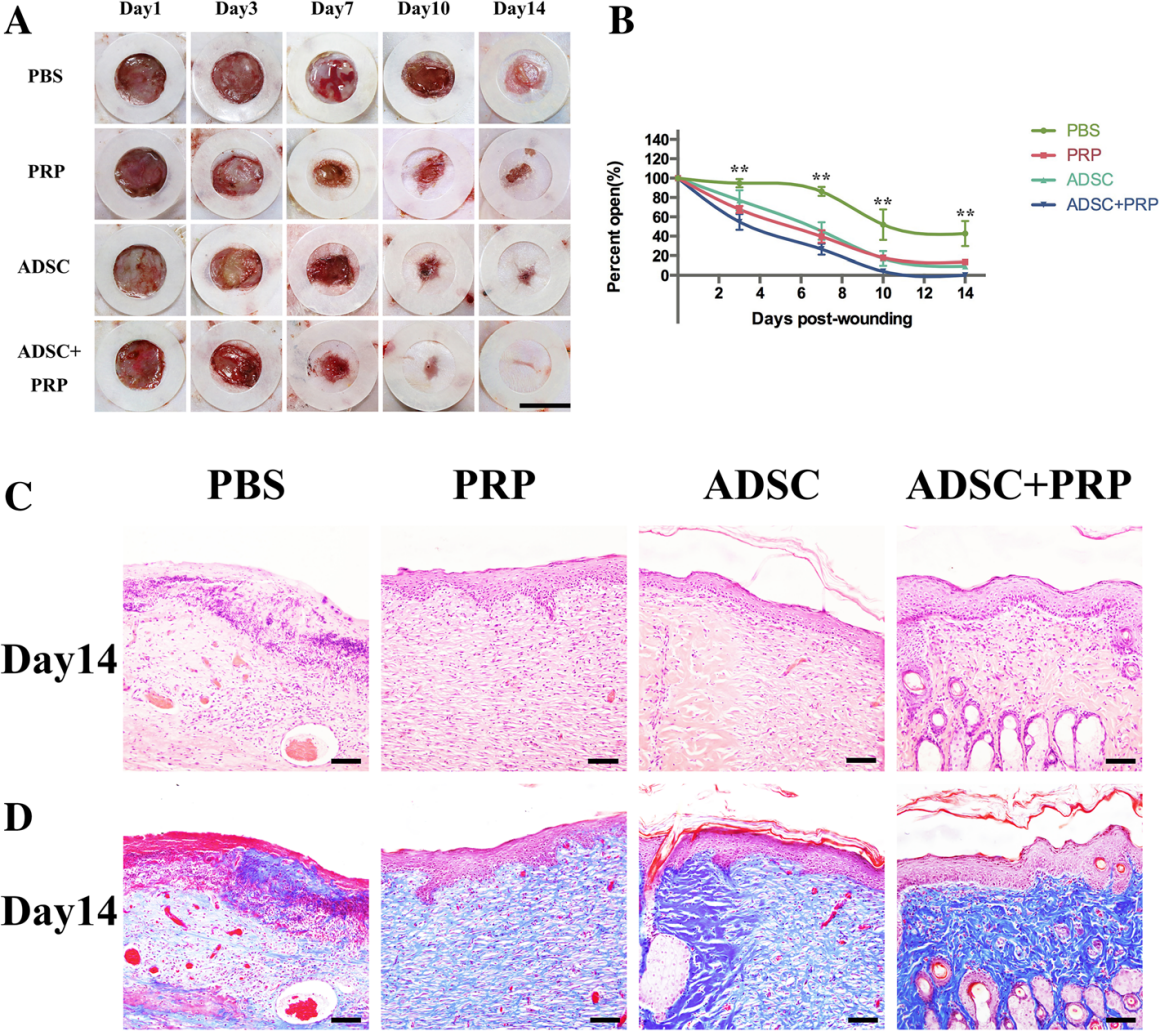
Wound healing in a diabetic rat excisional wound model and histology of wound beds posttreatment. **a** Representative images of the wounds show that compared to PBS, PRP, and ADSCs, treatment with ADSC+PRP accelerated wound healing, and full epithelialization was observed by day 10. Scale bar = 1 cm. **b** The wound closure percentage was evaluated postwounding. The ADSC+PRP group exhibited significantly accelerated wound healing compared to that exhibited by the other groups. Values were expressed as mean ± SEM (*n* = 12 animals per group, 24 wounds total per group). Statistics were performed using one-way ANOVA and showed statistically significant differences between groups, all six combinations of groups (PBS and PRP, PBS and ADSCs, PBS and ADSC+PRP, PRP and ADSCs, PRP and ADSCs+PRP, and ADSCs and ADSCs+PRP) were statistically significant with *p < 0.05 or better, as evaluated by Tukey post hoc test. **c** Hematoxylin and eosin staining on day 14 postwounding showed a thicker epidermis and increased appendages in the dermis of the ADSC+PRP group compared with the other groups. Scale bars = 50 μm. **d** Collagen deposition in the dermis was visualized with Masson’s trichrome staining and microscopy. The analysis generally demonstrated that compared to PBS, PRP, and ADSCs, treatment with ADSC+PRP promoted epithelialization. Scale bars = 50 μm (significance was set to *p < 0.05, **p < 0.01, and ***p < 0.001)

To determine the optimal concentration of PRP for combination with ADSCs, various concentrations of PRP were placed in the lower chamber of a Transwell plate to attract ADSCs cultured in the upper chamber. We found that 20% PRP induced the greatest migration of ADSCs, and this concentration of PRP was applied in this study (Fig. 2c). A diabetic rat model was successfully established, and full-thickness skin wounds were created on the backs of the model rats. Forty rats were randomly divided into 4 groups and injected with PBS, ADSCs, PRP, or ADSC+PRP. The areas of the cutaneous wounds were monitored every 3–4 days, and we found that compared with PBS, the application of ADSCs, PRP, or ADSC+PRP significantly promoted wound closure (Fig. 3a). It was obvious that the ADSC+PRP group exhibited that highest wound closure rate (Fig. 3b). Wound reepithelialization and matureness were assessed 14 days postinjury; compared to the other groups, the ADSC+PRP group exhibited a thicker epidermis and increased appendages in the dermis (Fig. 3c). The wounds injected with ADSC+PRP presented well-organized collagen deposition in the dermis, as determined by Masson’s trichrome staining (Fig. 3d).

### Combined treatment with ADSCs and PRP promoted new blood vessel formation in diabetic wounds

**Fig. 4:**
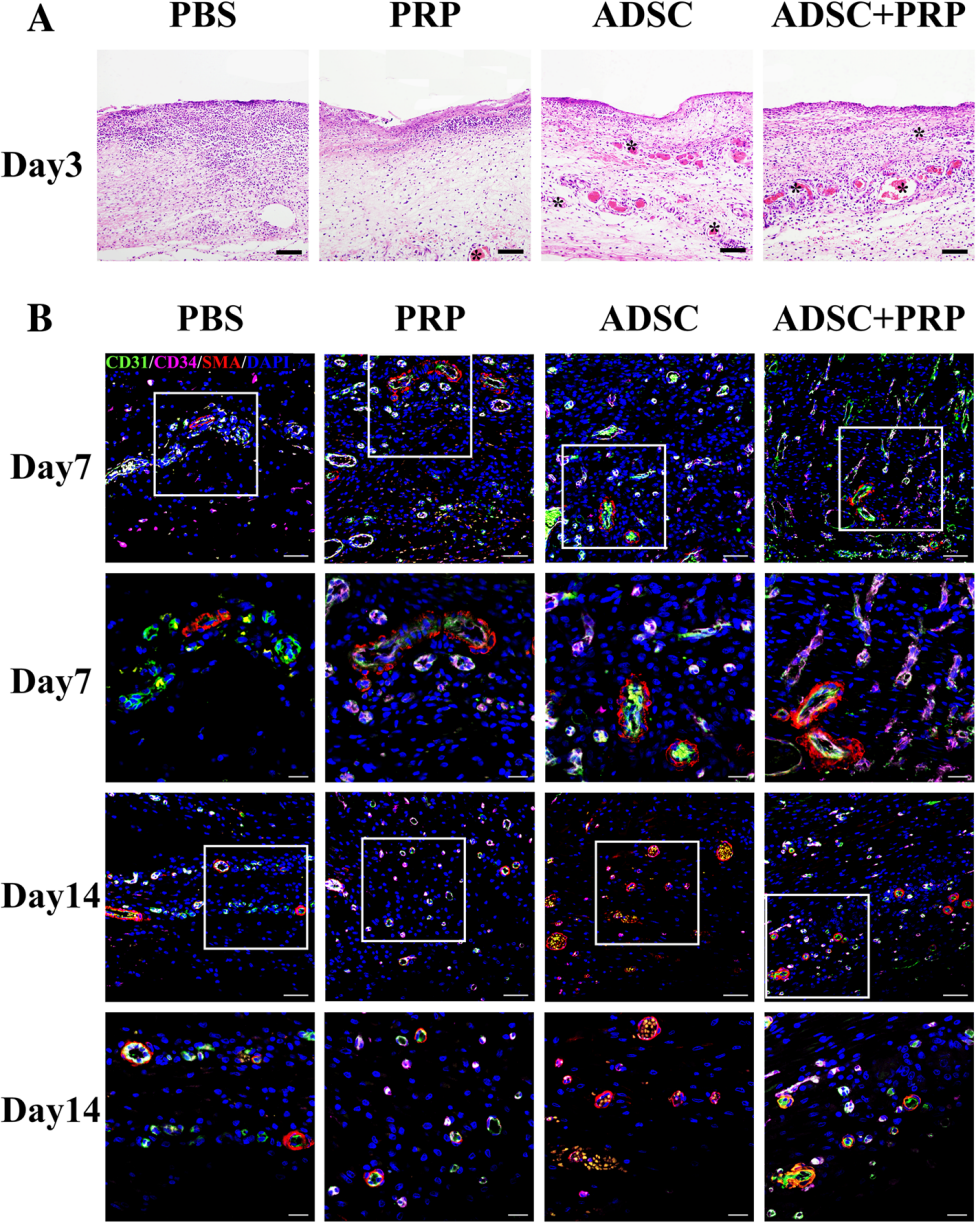
Evaluation of blood vessel formation. **a** Early angiogenesis in the wound beds was visualized on day 3 postwounding by hematoxylin and eosin staining. Staining showed an increased number of blood vessels forming in the wound in the ADSC+PRP group compared with the other groups. The black arrows indicate blood vessels. Scale bars = 50 μm. **b** Representative images of tissue sections from diabetic rats immunostained for CD31 (green), CD34 (pink), and α-SMA (red) and quantification of **c** the capillary density and the proportion of CD31+, CD34+, and α-SMA+ cells 7 and 14 days postwounding (× 200, scale bars = 50 μm; × 400, scale bars = 20 μm; *p < 0.05; *n* = 3 wounds per group)

We next explored early angiogenesis by hematoxylin and eosin staining and observed increased blood vessel formation in the wounds of the ADSC+PRP group compared to those of the PBS, ADSCs, and PRP groups (Fig. 4a). We further evaluated the expression of the vessel markers CD34, CD31, and α-SMA by immunohistochemistry. The wounds of the ADSC+PRP group exhibited the highest expression of CD34 and CD31, which indicated that ADSC+PRP treatment induced more new blood vessel formation. However, there was no significant difference in α-SMA expression among the groups (Fig. 4b). Taken together, our results suggested ADSC+PRP improved diabetic wound healing by promoting neovascularization.

### Combined treatment with ADSCs and PRP improved diabetic wound healing, and these improvements were related to angiogenesis and vasculogenesis

**Fig. 5:**
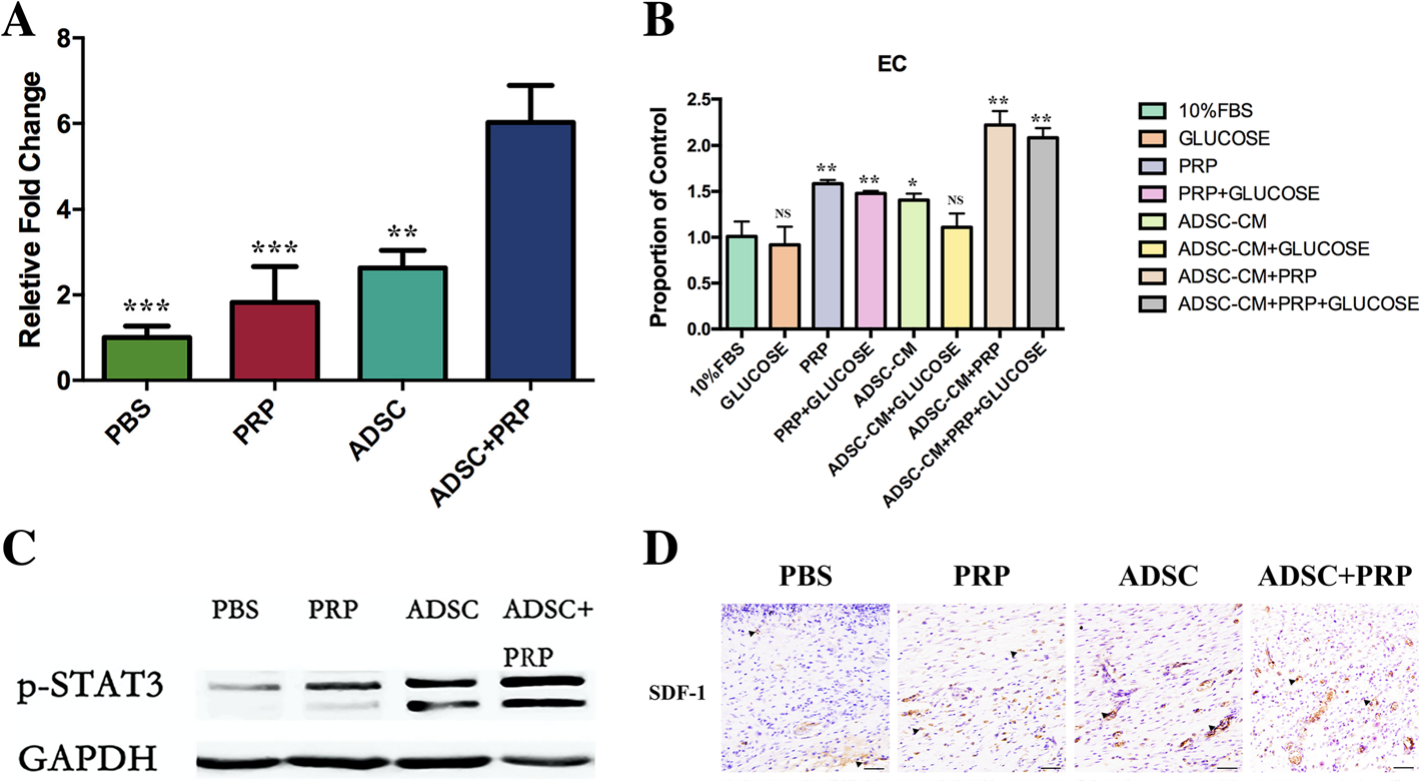
Effects of ADSC+PRP in promoting angiogenesis and vasculogenesis. **a** ELISA of VEGF in skin tissue lysates postwounding showed higher levels of VEGF protein production in the wounds of the ADSC+PRP group than those of the other groups. All experiments were performed in triplicate and were repeated three times to confirm the findings. Values were expressed as mean ± SEM. Statistical analysis was evaluated using one-way ANOVA and Tukey post hoc test (significance was set to *p < 0.05, **p < 0.01, and ***p < 0.001). **b** ADSC+PRP significantly enhanced the proliferation of endothelial cells compared with the ADSC-CM, PRP, or control group; the similar phenomenon was also presented in high-glucose medium. All experiments were performed in triplicate and were repeated three times to confirm the findings. Values were expressed as mean ± SEM. One-way ANOVA and Tukey’s post hoc test showed statistically significant differences overall between the eight groups (significance was set to *p < 0.05, **p < 0.01, and ***p < 0.001). **c** Protein expression levels of p-STAT3 on wound tissues were detected by Western blot analysis. **d** Representative images of tissue sections from diabetic rats immunostained for SDF-1 14 days postwounding (× 400, scale bars = 20 μm; *p < 0.05; *n* = 3 wounds per group)

Angiogenesis and vasculogenesis are two types of neovascularization. We found that the expression of the proangiogenic factor VEGF was higher in the diabetic wounds of the ADSC+PRP group compared with those of the other groups, which demonstrated that ADSC+PRP stimulated angiogenesis (Fig. 5a). Moreover, we analyzed the proliferation capacity of endothelial cells cultured with PBS, ADSC-CM, PRP, or a combination of ADSC-CM and PRP. We found that compared to the other treatments, the combination of ADSC-CM with PRP more strongly promoted the proliferation of endothelial cells. More importantly, this enhancement of endothelial cell proliferation was retained when a high-glucose medium was added (Fig. 5b). Western blot analysis was used to determine the effect of ADSC+PRP on angiogenesis-associated protein. The ADSC+PRP group significantly upregulated the expression of p-STAT3 after 7 days of treatment compared with the other groups (Fig. 5c). We finally evaluated vasculogenesis by detecting the expression of SDF-1 by immunohistochemistry. The results showed that combined treatment with ADSCs and PRP induced a greater increase in the protein expression of SDF-1 than the other treatments (Fig. 5d).

## Discussion

Wound healing is a complicated but highly regulated biological process. Many factors can disturb the well-organized process, resulting in chronic or nonhealing wounds. Due to its molecular pathogenesis, diabetes is a factor that can disrupt the process of wound healing and lead to the development of a chronic or nonhealing wound. A series of studies have uncovered the probable pathophysiology of diabetic wounds, which includes decreased growth factor production, impaired angiogenesis, and defective cellular functions in the impaired wound [26]. The current conventional treatments, such as application of dressings, negative pressure therapy, autologous skin graft, and hyperbaric oxygen therapy, have several limitations and moderate efficacy [7]. Developing an innovative and more efficient treatment is urgent.

Stem cell therapy has become a promising approach to promote wound healing. Stem cells can not only secrete various growth factors but also directly transdifferentiate into vascular components or skin cells to correct the pathogenesis of diabetic wounds [6]. The clinical use of human allogenic ADSC transplant had been used for many years, while the safety and efficacy were confirmed in osteoarthritis, acute ischemic stroke, myocardial infarction, and so on [27]. ADSCs even have the possibility to be used in patients with COVID-19 pneumonia [28]. For chronic wounds, several mechanisms of ADSCs were described in each process of wound healing, including the reduction of inflammation, induction of angiogenesis, and promotion of fibroblast and keratinocyte growth [29].

PRP is another effective agent for wound healing because it contains abundant growth factors and other substances that can restore growth factors in diabetic wounds. The cellular processes, such as chemotaxis, cell differentiation, and angiogenesis, and several target cells in wound healing were stimulated by PRP [30, 31]. The antiapoptotic effect of PRP through the activation of the Bcl-2 protein and Akt signaling was a contributing factor to improve wound healing by stimulating hair growth [32]. Recently, the combined use with PRP and hyaluronic acid as a bio-functionalized scaffold showed many advantages for wound healing [30]. Similarly, PRP can act as a biomaterial scaffold for ADSCs, and furthermore, it can induce the development of angiogenesis for delivering proper nutrient and oxygen levels to grafted ADSCs [33]. Therefore, combining ADSCs with PRP might be an optimal therapeutic strategy for enhancing the healing of chronic/nonhealing wounds.

In this study, we established a wound model in diabetic rats to explore the treatment efficacy of a combination of ADSCs and PRP. We found that the ADSC+PRP group had a higher wound closure rate than the control, PRP, and ADSCs groups. Treatment with ADSC+PRP induced complete closure of diabetic wounds after 10 days, while the other treatments did not, indicating the synergistic effects of ADSCs and PRP. We observed histological changes in the wounds to evaluate the function of ADSCs and PRP and found that thicker epidermis and more well-organized collagen deposition were presented in the dermis. The stimulatory effects of ADSCs and PRP on proliferation and migration of fibroblasts and keratinocytes revealed the mechanism of the above phenomena [20].

In addition, more vascular structures appeared in the wounds of the ADSC+PRP group. New vessel formation, which allows delivery of nutrients and maintenance of oxygen homeostasis, is required for efficient wound healing. However, the crucial process of wound healing is impaired in diabetes mellitus. Diabetic hyperglycemia can impede angiogenesis by perturbing the balance of vessel growth and inducing the dysfunction of endothelial cells [34]. Defective new blood vessel formation is a major contributor to chronic/nonhealing wounds caused by diabetes. The ADSC+PRP-induced restoration of vessel formation in chronic wounds showed the potential to correct the abnormal healing process. We next verified the promoting effect of ADSC+PRP on new vessel formation by using immunofluorescence and found that the expression of the vascular markers CD31 and CD34 in the wounds was highest in the ADSC+PRP group. These results suggest that the therapeutic effect of ADSC+PRP is related to neovascularization in diabetic wounds.

An adequate newly developed vascular system is essential for wound healing. Angiogenesis is the primary method by which new blood vessels are developed and is tightly controlled by the balance of proangiogenic and antiangiogenic factors [35]. VEGF is one of the most important proangiogenic factors and can stimulate multiple steps of angiogenesis, including vasodilation, basement membrane degradation, endothelial cell migration, and proliferation [36]. The VEGF content in wounds was reduced in diabetic wounds compared to normal wounds, and this reduction in VEGF expression ultimately impaired angiogenesis [37]. Interestingly, we found that the combined application of ADSCs and PRP elevated the level of VEGF in diabetic wounds. The activation of endothelial cells in angiogenesis enhanced the capillary structure. Therefore, we speculated that ADSC+PRP also has a positive effect on the proliferation of endothelial cells. The paracrine activity of ADSCs mediated by ADSC-CM is an important way in which ADSCs interact with other cells in the extracellular microenvironment. We used ADSC-CM, PRP, or both to stimulate the growth of endothelial cells in vitro and found that the combination of the two treatments had the strongest effect on increasing the proliferation of endothelial cells. The stimulation of endothelial cell proliferation also persisted under high-glucose conditions. This phenomenon might partly explain the increased vessel formation in diabetic wounds treated with ADSC+PRP. It has been established that the induction of VEGF expression is regulated by active STAT3 in the spectrum of physiological and pathological conditions [38]. So we detected the p-STAT3 expression in the diabetic wound and found ADSC+PRP could increase the expression of p-STAT3. Therefore, it is reasonable to presume that ADSC+PRP might enhance angiogenesis through STAT3/VEGF axis. It is also worth noting that VEGF is not limited to accelerating angiogenesis but also affects other cells, such as fibroblasts and keratinocytes [39], to contribute to wound healing.

In addition to angiogenesis, vasculogenesis also contributes to neovascularization. When injury occurs, endothelial progenitor cells (EPCs) are mobilized from the bone marrow into the bloodstream and then recruited to the hypoxic environment of the wound. The above processes, called EPC mobilization and homing, are impaired in patients with diabetes [26]. The mobilization of EPCs could be partly stimulated by hyperoxia, but the inducer of EPC homing, SDF-1, is indeed decreased in patients with diabetes [40]. Gallagher found that SDF-1 injection into diabetic wounds partially reverses the defect in EPC recruitment and ultimately enhances diabetic wound healing [40]. We were surprised to find that SDF-1 expressed in diabetic wounds was restored by ADSC+PRP treatment.

**Fig. 6:**
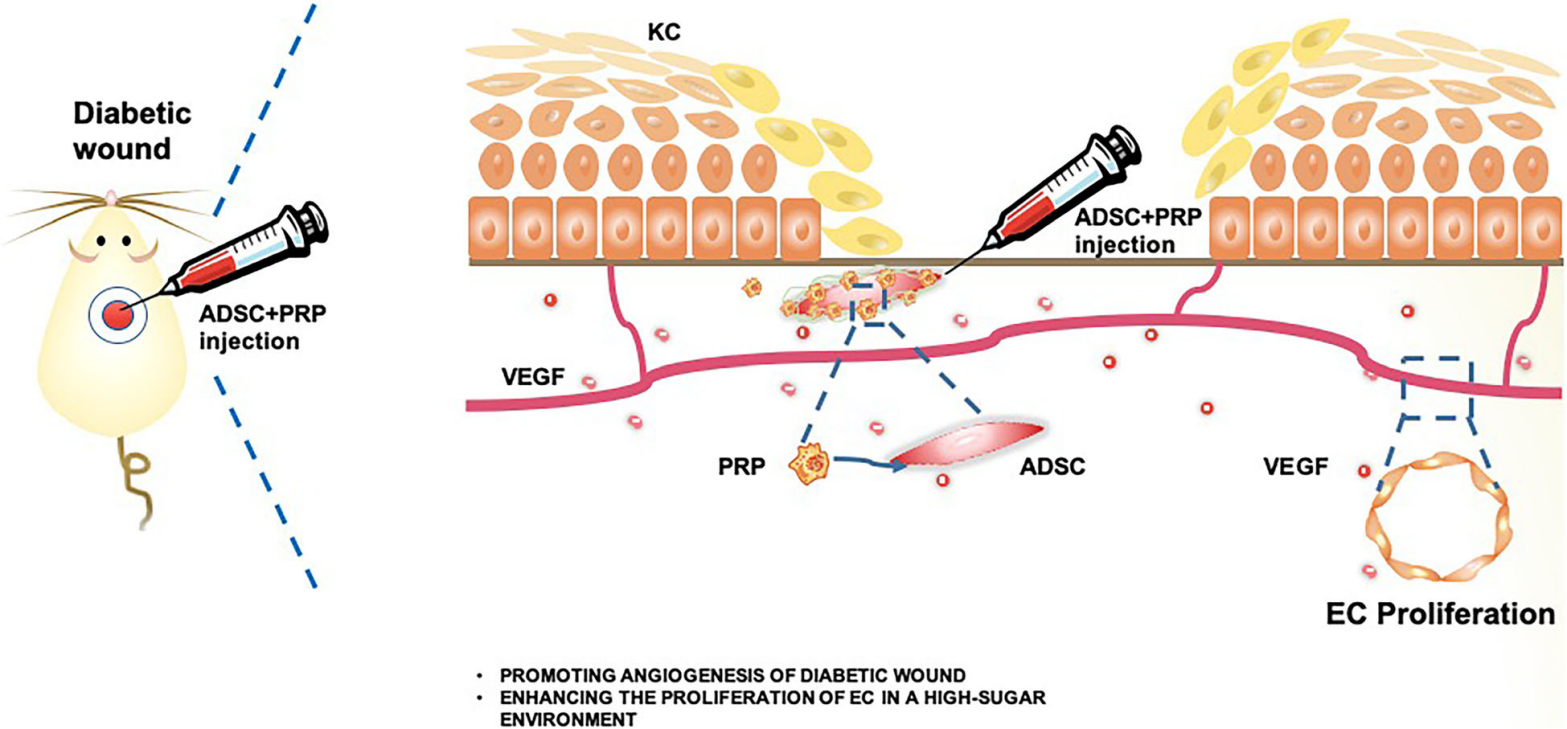
Graphical abstract. Graph showing the combined application of ADSCs and PRP could enhance diabetic wound healing

Improvements in diabetic wound healing are urgently needed. Here, we propose that the combined application of ADSCs and PRP can enhance diabetic wound healing through neovascularization and is therefore an optimal treatment for diabetic wounds (Fig. 6).

## Conclusions

In summary, this study reveals that the combination of ADSCs and PRP can optimally enhance diabetic wound healing. The underlying mechanism involves neovascularization. This study provides an innovative collaborative scenario and a strong foundation for further treatment of wound healing.

## Data Availability

Not applicable.

## Abbreviations

ADSCs: Adipose-derived stem cells
PRP: Platelet-rich plasma
PDGF: Platelet-derived growth factor
TGF: Transforming growth factor
VEGF: Vascular endothelial growth factor
IGF: Insulin-like growth factor
SD: Sprague-Dawley
PBS: Phosphate-buffered saline
LG-DMEM: Low-glucose Dulbecco’s modified Eagle medium
FBS: Fetal bovine serum
FITC: Fluorescein isothiocyanate
SDF-1: Stromal cell-derived factor-1
DAB: 3,3′-Diaminobenzidine
DAPI: 6-Diamidino-2-phenylindole
ELISA: Enzyme-linked immunosorbent assay
ADSC-CM: Adipose-derived stem cell culture medium
MTS: 3-(4,5-Dimethylthiazol-2-yl)-5-(3-carboxymethoxyphenyl)-2-(4-sulfophenyl)-2H-tetrazolium
MSC: Mesenchymal stem cell
EPCs: Endothelial progenitor cells
